# Expanding the Applicability of Poly(Ionic Liquids) in Solid Phase Microextraction: Pyrrolidinium Coatings

**DOI:** 10.3390/ma10091094

**Published:** 2017-09-18

**Authors:** David J. S. Patinha, Liliana C. Tomé, Mehmet Isik, David Mecerreyes, Armando J. D. Silvestre, Isabel M. Marrucho

**Affiliations:** 1 Instituto de Tecnologia Química e Biológica António Xavier, Universidade Nova de Lisboa, Av. Da República, 2780-157 Oeiras, Portugal; davidpatinha@itqb.unl.pt (D.J.S.P.); liliana.tome@itqb.unl.pt (L.C.T.); 2 CICECO—Aveiro Institute of Materials and Department of Chemistry, University of Aveiro, 3810-193 Aveiro, Portugal; 3 POLYMAT, University of the Basque Country UPV/EHU, Joxe Mari Korta Center, Avda. Tolosa 72, 20018 Donostia-San Sebastian, Spain; isik.mehmet@ehu.eus (M.I.); david.mecerreyes@ehu.es (D.M.); 4 IKERBASQUE, Basque Foundation for Science, E-48011 Bilbao, Spain; 5 Centro de Química Estrutural, Instituto Superior Técnico, Universidade de Lisboa, Avenida Rovisco Pais, 1049-001 Lisboa, Portugal

**Keywords:** solid phase microextraction, poly(ionic liquids), UV-photopolymerization, gas chromatography, steel coatings, fibers

## Abstract

Crosslinked pyrrolidinium-based poly(ionic liquids) (Pyrr-PILs) were synthesized through a fast, simple, and solventless photopolymerization scheme, and tested as solid phase microextraction (SPME) sorbents. A series of Pyrr-PILs bearing three different alkyl side chain lengths with two, eight, and fourteen carbons was prepared, characterized, and homogeneously coated on a steel wire by using a very simple procedure. The resulting coatings showed a high thermal stability, with decomposition temperatures above 350 °C, excellent film stability, and lifetime of over 100 injections. The performance of these PIL-based SPME fibers was evaluated using a mixture of eleven organic compounds with different molar volumes and chemical functionalities (alcohols, ketones, and monoterpenes). The Pyrr-PIL fibers were obtained as dense film coatings, with 67 μm thickness, with an overall sorption increase of 90% and 55% as compared to commercial fibers of Polyacrylate (85 μm) (PA85) and Polydimethylsiloxane (7 μm) (PDMS7) coatings, respectively. A urine sample doped with the sample mixture was used to study the matrix effect and establish relative recoveries, which ranged from 60.2% to 104.1%.

## 1. Introduction

Solid phase microextraction (SPME) is a solventless sampling and preconcentration technique that has gained popularity since its introduction by Pawliszyn and co-workers in the early 1990s [1]. This technique combines the sampling, extraction, concentration, and sample introduction into the apparatus steps in a single micro device [2]. The corner stone of this device is the polymeric coating material, which allows for the sorption/desorption of the compounds of interest. Polydimethylsiloxane (PDMS), polyacrylate (PA), and divinylbenzene (DVB) [3] are the most commonly used commercially available coatings. However, and despite their many advantages, these coatings might restrict and hinder the detection and analysis of certain compounds due to their lack of specificity. To overcome this problem, a broad range of alternative phases, spanning from carbon-based nanomaterials [4], metal-organic frameworks [5], biomaterials [6], and ionic liquids [7], have been recently studied and evaluated.

Ionic liquids (ILs) have emerged as promising coating materials for SPME due to their unique properties: besides their almost null vapor pressure and low flammability, the most advantageous feature of ILs is their chemical tunability [8], which can be attained through a plethora of cations and anions that can be appropriately chosen to prepare task-specific ILs. This is particularly attractive in the development of highly specific SPME fiber coatings, since the number of cation-anion combinations can be, theoretically, as high as 10^18^ [9]. ILs in SPME coatings were introduced by Liu et al. [10] in 2005. The authors prepared a 1–octyl–3–methylimidazolium hexafluorophosphate ([C_8_mim][PF_6_]) coating to analyze a mixture of aromatic compounds present in paints. Despite their innovative use, the proposed fiber coatings were less sensitive than the commercial ones, probably due to the small amount of IL used. Another drawback of using ILs in SPME fiber coatings is their significant decrease in viscosity with temperature, resulting in a loss of coating and a consequent need of recoating before each extraction. In this line, poly(ionic liquids) (PILs), a sub-group of polyelectrolytes [11] that combines the mechanical and thermal properties of polymers with the high tunability and versatility of ILs, have been considered as more promising alternatives in this type of analyte-selective application [12,13,14]. Analytes such as polycyclic aromatic hydrocarbons [15], CO_2_ [16], alcohols and amines [17,18], and benzene derivatives among others [19,20] have been the focus of SPME analysis using PILs coatings. The good results, both in terms of stability and performance, confirm the real advantage of using PILs as SPME coatings. However, all of these studies are restricted to imidazolium-based polycations [21], and therefore the most appealing feature of these materials, their tunability, still remains totally underexplored. Considering the increasing challenges faced by analytical chemistry in a wide range of domains, such as for example, environmental, food, and health sciences, the development of new SPME coating material clearly deserves a deeper investigation. Consequently, we here unravel the effect of changing the polycation in PILs-based SPME fibers, moving from imidazolium-based polymers to pyrrolidinium-based polymers. Similarly to ionic liquids, where the choice of the cation-anion combination leads to improved performances, PILs tunability can be crucial for this type of highly specific tasks.

In this work, we present the use of pyrrolidinium-based PILs (Pyrr-PILs) bearing different alkyl chain lengths (C2, C8, and C14) as SPME fiber coatings. Although the excellent properties of these PILs in gas separation processes have been formerly demonstrated [22], their synthesis called upon complex high temperature processes and nasty solvents [23]. With the intent to improve the film processability, as well as the sustainability of the synthetic process, we herein use photopolymerization, a fast, low temperature, and solvent free process based on a methodology proposed by Zeng et al. [24]. Also, in order to increase the mechanical and thermal properties of the obtained coatings, a crosslinker was added to obtain a 3D polymeric network. Moreover, the SPME fibers here presented combine the two usual steps of polymer synthesis and fiber preparation in a single step, abducting costly and time consuming polymer cleaning and fiber coating preparation steps. The final Pyrr-PILs with three alkyl side chains of different sizes (C2, C8, and C14) were chemically and thermally characterized before their use as SPME fibers. Finally, a mixture composed of alcohols, ketones, and monoterpenes was used to evaluate the performance of the prepared Pyrr-PIL SPME fibers. The chosen molecules, such as 1–butanol, 2–hexanone, or (*S*)-(−)-β-citronellol, are widely used in flavor, perfume, or food industries, and their presence in human fluids has been related with different types of cancer [25]. Thus, a sample of human urine was used to evaluate the matrix effect and perform recovery tests.

## 2. Results and Discussion

### 2.1. Synthesis of Ionic Liquid Monomers and Poly(Ionic Liquids)

**Figure 1:**
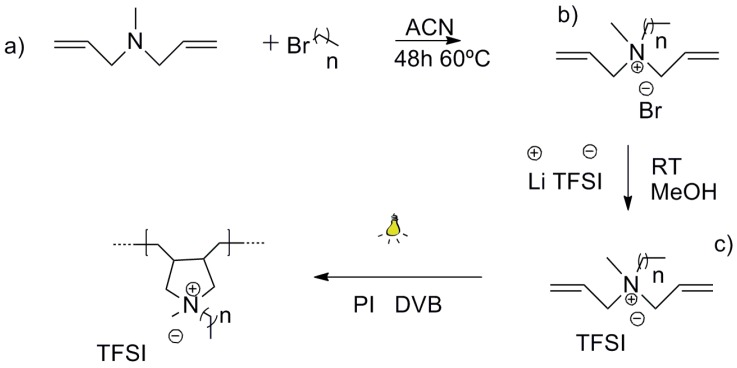
Overall synthetic scheme of pyrrolidinium-based poly(ionic liquids) (Pyrr-PIL) used in this work: (**a**) synthesis of the quaternary ammonium monomers with different alkyl chain lengths; (**b**) anion exchange reaction step to obtain the IL monomers with poly(methyltetradecyl pyrrolidinium) (TFSI) anion; and, (**c**) photopolymerization process.

Quaternary ammonium monomers with varying alkyl chain lengths were synthesized through the quaternization of the commercially available diallylmethyamine (DAM) compound (Figure 1a). The quaternary ammonium monomers were obtained with high purity, with yields higher than 90% in all cases. ^1^H-NMR spectra confirmed the successful attachment of the alkyl groups that displayed methyl -CH_3_ resonances around 0.7 ppm, 0.9, and 1.3 ppm for [DAMC_14_], [DAMC_8_] (Figure S1), and [DAMC_2_] cations, respectively. The adjacent methylene -CH_2_- resonances were observed between 1.01–1.37 ppm, 1.20–1.40 ppm, and 2.85–2.95 ppm for [DAMC_14_], [DAMC_8_] and [DAMC_2_] cations, respectively. Due to the specific requirements of the SPME process, specifically the high temperature of the injector (200–280 °C) and the thermal stability of the materials used in the fiber preparation is of great importance. It is well known that, due to their high basicities, halide anions decrease the thermal stability of ionic materials through elimination mechanisms resulting in the formation of unsaturated groups and free amines [26]. Therefore, in order to improve the thermal stability of the IL-based materials, bulky fluorinated anions were used [12,27]. Thus, anion exchange metathesis reactions were performed over the synthesized quaternary ammonium monomers to replace the halide anion with bis(trifluoromethanesulfonyl)imide ([TFSI]^−^) (Figure 1b). In all three cases, the resulting monomers were viscous liquids at room temperature, which allowed us to perform facile photopolymerization reactions without the use of complex synthetic strategies, which require temperature and purification steps. In all of the cases, a desired amount of crosslinker was mixed with the IL monomer to improve the structural integrity of the fiber material. A calculated amount of photoinitiator was added into the mixture prior to exposure to UV light. The reaction steps to obtain the monomers and the ensuing polymers are depicted in Figure 1.

The time required for complete polymerization of the [DAMC*_n_*][TFSI] monomers was determined by means of ^1^H-NMR, through the disappearance of the resonances attributed to the polymerizable groups. For that purpose, a mixture composed of each one of the monomer of interest and 5% in weight relative to monomer of 2-hydroxy-2-methylpropiophenone as photoinitiator (PI) was prepared and stored at 4 °C. Each mixture was exposed to UV light for exposure times varying from 10 to 60 s. It was observed that after 60 s of exposure, all of the experiences resulted in high degrees of monomer conversion. 60 s were then used to prepare crosslinked materials by adding DVB to the reaction mixture. In order to quantify any possible unreacted monomer in the crosslinked polymer, a sample of each crosslinked material was left in acetone so that any unreacted monomer was extracted. The insoluble parts were filtered, the acetone was dried, and deuterated solvent was added. The resulting ^1^H-NMR spectra did not show any signs of the [DAMC*_n_*][TFSI] monomers and DVB, meaning that complete polymerization was successfully achieved.

Despite the presence of two polymerizable groups in each monomer, no crosslinking was observed when exposing only the monomer and photoinitiator to the UV light. The five-side ring closing mechanism [28,29], is preferred in both free radical polymerization and photopolymerization, and consequently only the pyrrolidinium ring is formed.

### 2.2. Thermal Characterization of the Poly(Ionic Liquids)

**Figure 2:**
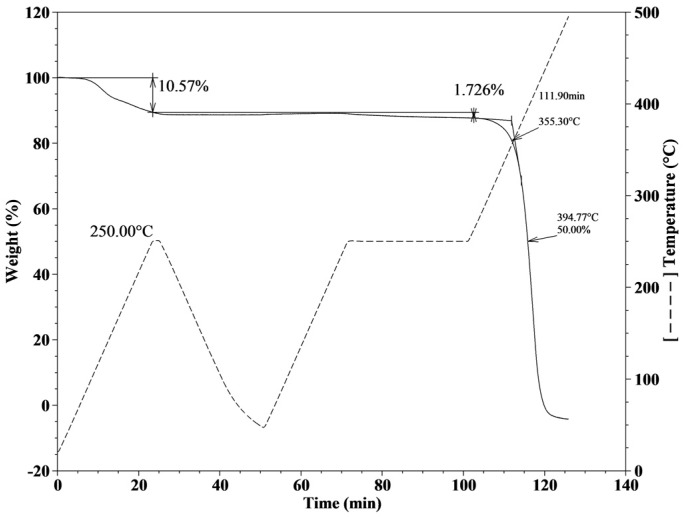
TGA thermogram of crosslinked poly(methyltetradecyl pyrrolidinium) (TFSI) (pD14).

The thermogravimetric analysis (TGA) plot of the crosslinked poly(methyltetradecyl pyrrolidinium) (TFSI) (pD14) film is presented in Figure 2. These experiments allow for accessing the thermal stability of the polymer, while they also mimic the polymer annealing and the fiber conditioning steps. In a first stage, the material was subjected to an increase in temperature to 250 °C at 10 °C/min, and then cooled down outside of the oven, allowing for the long alkyl side chains reorganization. Also, some of non-stable polymer fractions, as well as some unknown impurities and solvents trapped in the material, are released, as it can be seen by the weight loss of about 10% during the first 25 min. This step is vital for fiber coating reproducibility. From this point onwards, the material is considered to be prepared and the second stage mimics the fiber conditioning at the GC injector. The objective in the second stage is to evaluate the material thermal stability by placing it at a high temperature (250 °C) for a long period of time (30 min). As it can be seen in Figure 2, for the pD14 sample, the weight loss is about 1–2% during the whole process, indicating the thermal stability of the materials. The third and final stage of the experiment allows for the determination of the decomposition temperature of the material, which showed an onset temperature of about 355 °C and 50% of the initial weight loss at 394 °C. Regarding the other two crosslinked polymers, namely poly(methyloctyl pyrrolidinium) TFSI (pD8) and poly(methylethyl pyrrolidinium) TFSI (pD2), the results are available the Figures S2 and S3, respectively. In fact, the behavior of these three polymeric materials is very similar. In the first stage, the pD2 loses 9% of weight, while pD8 about 5%. Then, in the second stage, the weight loss is between 0.8% and 1.8% for the three polymeric materials. In the third and final step, the decomposition temperature (*T_d_*) decreases with increasing the alkyl side chain length, from 368 °C for pD2, 361 °C for pD8, and 355 °C for pD14 (Table S1).

The results of differential scanning calorimetry (DSC) analysis of crosslinked pD14 film are shown in Figure S4. Since pD14 is a crosslinked material, thus an amorphous structure, no melting is expected [30]. However, the presence of glass transition temperature (*T_g_*) is very helpful when developing new materials for SPME since above this temperature the rubbery state is attained, allowing for an easier sorption of the analytes into polymer coating [31]. A *T_g_* can be observed around −21 °C, confirming the rubbery state of the coating at operation temperatures. The *T_g_*’s observed in the DSC´s third temperature cycle of the three materials under study fall in the same region, between −16 °C and −23 °C (Table S1 and Figure S5), thus rendering a large range of working temperatures for these coatings, both in the sorption and desorption steps.

### 2.3. Fiber Coating Analysis

**Figure 3:**
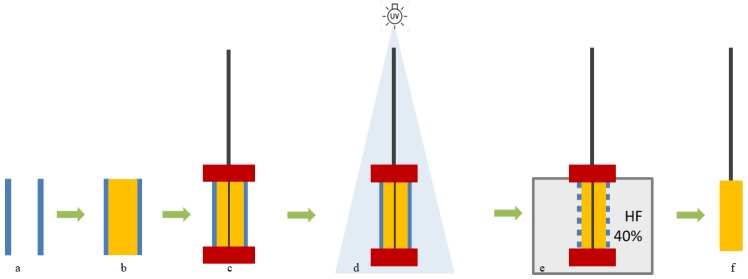
Representation of the fiber coating procedure using a virtual longitudinal cut at the center of the material; (**a**)—empty ST; (**b**)—ST filled with M1; (**c**)—two red rubber stoppers were placed at the ends of the ST with steel wire in the middle; (**d**)—UV polymerization step; (**e**)—ST is destroyed by HF; and, (**f**)—final material coated on top of the steel wire.

The fiber fabrication is described in detail in Section 3.3 and is depicted in Figure 3. Basically, a mixture (M1) of monomer, photoinitiator, and crosslinker was inserted in a silica tube (ST), and polymerized under UV—light for 60 s. Afterwards, the silica tube was destroyed by immersion in hydrofluoric acid (HF) during 1 h. The whole procedure takes about 2 h to finish.

**Figure 4:**
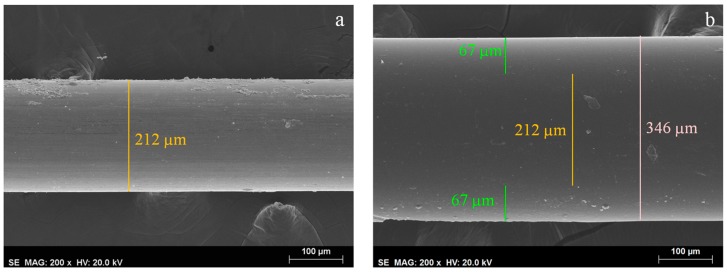
SEM images of: (**a**) untreated steel wire with a magnification of 200× (average outer diameter of 212 μm); (**b**) steel wire coated with crosslinked poly(methyltetradecyl pyrrolidinium) TFSI with a magnification of 200× (average outer diameter of 346 μm).

In Figure 4, the SEM images of the bare steel wire (Figure 4a) and the coated steel wire (Figure 4b) show a very homogeneous surface coating with a regular thickness of 67 μm, with a total volume of 0.561 mm^3^ of coating material, which is more than PDMS7 (0.026 mm^3^) and similar to PA85 (0.521 mm^3^) [32]. This length and thickness can be changed by using silica tubes with different sizes and different internal diameters, maintaining the homogeneity of the overall fiber that will result in a better reproducibility. Moreover, this method allows for the production of almost identical fibers from batch to batch that result in a high repeatability between different fibers.

### 2.4. Optimization of Headspace-SPME Parameters

In SPME, the optimization of parameters at both the sorption and desorption stages is crucial to achieve the optimal results. To maximize sensitivity and reproducibility, variables such as temperature, time, stirring rate, pH, ionic strength, and ratio of volume of the sample to volume of the headspace are commonly optimized for each matrix.

#### 2.4.1. Sorption Time

**Figure 5:**
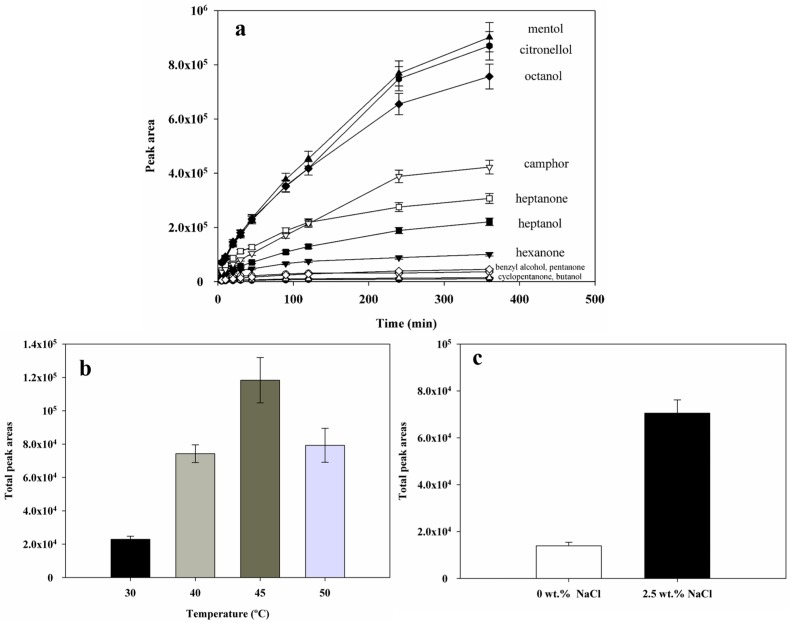
Effects of the different extraction conditions on the efficiency of pD14 towards standard solutions. Extraction time (**a**) (*T* = 40 °C, 2.5 wt % of NaCl, 200 rpm, concentration: 50 mg·L^−1^); extraction temperature (**b**) (*t* = 15 min, 2.5 wt % of NaCl, 200 rpm, concentration 20: mg·L^−1^) and salt concentration (**c**) (*T* = 45 °C, *t* = 15 min, 200 rpm, concentration: 2 mg·L^−1^).

The sorption versus time profiles of each compound present in the probe mixture for the pD14 fiber are depicted in Figure 5a. It can be observed that not all compounds reached the sorption equilibrium after 360 min. This type of behavior was reported before for different matrixes [33] and it is usually attributed to the thickness of the fibers. In the present case, the 67 μm thickness coating resulted in a long equilibration time. Thus, a compromise was made and 15 min of headspace extraction time was used in the experiments.

#### 2.4.2. Extraction Temperature

Temperature plays a crucial role in the partition of the analytes in liquid phase—vapor phase—fiber coating equilibria, and therefore greatly affects the sensitivity of the technique [34]. Actually, temperature can have opposite effects: in headspace sampling, higher temperatures promote analytes to move from the aqueous phase to the headspace, thus increasing the mass transfer rates into the fiber [35]; on the other hand, higher temperatures can decrease the distribution coefficient of the analyte from the fiber coating material back again to the headspace, decreasing the amount extracted [35]. In this work, the influence of extraction temperature in the interval between 30 °C and 50 °C was studied. The results obtained are shown in Figure 5b, where an increase in the total peak area is observed until 45 °C, followed by a marked decrease for higher temperatures, in agreement with previous works [35,36]. Therefore, and since no differences in behavior were detected for individual compounds, a temperature of 45 °C was taken as the optimal extraction temperature.

#### 2.4.3. Salt Addition

The addition of modifiers, such as salts, can induce the well-known salting-out effect, since there is a competition for the water molecules between the salt and the analytes, which is usually won by the salts due to their coulombic nature. In Figure 5c, the confirmation of the influence of the salt concentration (0 and 2.5 wt %) in the total peak areas is presented. Comparing the results without and with the addition of salt, a five-fold increase in the total peak area can be observed. Since the used NaCl concentration (2.5 wt %) is far from saturation (26 wt % at 25 °C), the salt effect can be further adjusted if needed depending also on the compound water solubility.

### 2.5. Performance of the Prepared Fibers

**Figure 6:**
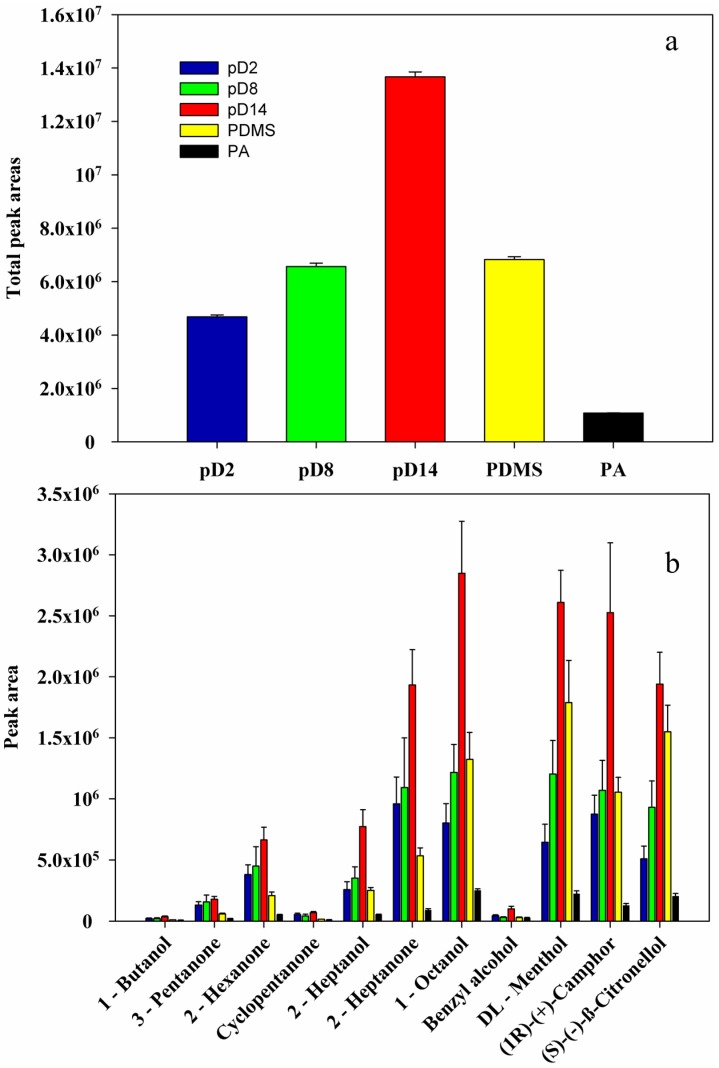
Comparison and performance of the prepared fibers along with commercial fibers in terms of: (**a**) total detected areas and (**b**) individual compound area. (*T* = 45 °C, *t* = 15 min, 2.5 wt % of NaCl, 200 rpm, concentration: 200 mg·L^−1^).

After the optimization of the SPME parameters to attain a maximum of total peak areas for pD14, extractions were carried out with the other two fibers (pD2 and pD8) at these fixed conditions. The extraction efficiencies for each fiber and the total detected areas are presented in Figure 6a, along with the results obtained for the commercial PDMS7 and PA85 fibers under the same experimental conditions. Since our standard solution is a mixture of polar and semi-volatile compounds, but it also contains rather less polar compounds with relatively long alkyl chains, such as in 1-octanol or 2-heptanone, two commercial fibers were chosen to establish comparisons with our homemade fiber: PA85, that is a suitable and recommended SPME phase for polar and semi volatiles, and PDMS7, that is a SPME phase recommended for non-polar molecules with molecular weights of 125 up to 600. More complex commercial SPME fibers, such as PDMS/DVB or others, were avoided since we want to establish a fair comparison with other pure polymers.

An increase in the overall extraction efficiency can be observed with the increase of the alkyl chain length, from pD2 to pD14. This behavior can be attributed to a more packed structure obtained for pyrrolidinium-based PILs with shorter chains, yielding a material with smaller free volume [37]. Conversely, for a material with longer alkyl chain lengths, the free volume increases, and therefore, the analytes can better accommodate, diffuse, and penetrate into the material resulting in superior extraction efficiencies. The increase of the alkyl chain length does not seem to have much effect on the extraction of polar compounds, such as 1-butanol, where similar peak areas were obtained for pD2, pD4, and pD14. In terms of total detected peak areas, pD14 presents 56% and 68% higher efficiency than pD8 and pD2, respectively. From the obtained results shown in Figure 6a, it can be observed that the overall pD14 fiber showed the best results, with a 55% higher total peak area than the PDMS fiber with 7 μm and more than 90% total peak area higher than the PA fiber with 85 μm. Apart from the different chemical nature of each polymer, the difference in the film thickness between our coatings (67 μm) and the commercial coatings used here PDMS (7 μm) and PA (85 μm) is an important factor affecting the efficiency of our fibers since increasing the film thickness can lead to longer extraction times, due to a slower mass transfer. Thus, the construction of a thinner PIL coating will fasten the mass transfer in the desorption step, therefore, possibly increasing the performance of the PIL fibers. Moreover, it is known that fibers that are vitreous solids at room temperature, such as the PA fiber, take more time to sorb analytes unless high temperatures are used.

In terms of individual compounds (Figure 6b), similar sorption profiles were observed for the three prepared fibers for all of the components in the prepared mixture, probably due to the small change in the materials properties owing to the lack of functional groups in the alkyl side chains of the prepared materials. However, it is notorious that the sorption of analytes with longer chains, such as 1-octanol, increases with the increasing of the alkyl chain length in our material, in agreement with what was said before for the increase in the free volume with the increase of the alkyl chain length. On the other hand, molecules such as cyclopentanone or benzyl alcohol do not show much affinity towards any of the prepared fibers.

To better understand these behaviors, properties such as the vapor pressure (*V_P_*), molar volume (*V_M_*), and water solubility (*W_S_*) of each compound must be taken into account. First, higher water solubility will obstruct the extraction from the aqueous phase to the headspace. Second, the higher the molar volume, the less absorption within the polymer is achieved. In the used mixture, the molecules with higher *V_P_* are ketones, followed by alcohols, and later monoterpenes. In terms of *V_M_*, (*S*)-(−)-β-citronellol, 1-octanol, 2-heptanone, and 2-heptanol show the largest values. Regarding *W_S_*, values lower than 2 g·L^−1^ are found for 1-octanol and the three monoterpenes studied, in the range of (2–15) g·L^−1^ for the compounds 2-heptanone, 2-heptanol, 2-hexanone, and cyclopentanone, and only with *W_S_* higher than 43 g·L^−1^, there are benzyl alcohol, 3-pentanone, and 1-butanol. From Figure 6b, it can be observed that the peak areas detected for each compound are closely related to their *W_S_*. Moreover, it can also be concluded that after *Ws*, *V_M_* also influences the most detected compounds, which are those with the largest molar volumes, such as 2-heptanone and 2-heptanol. These two facts point out that this fiber is the more suitable to detect a wide range of compounds, from bulky non-polar compounds to small polar molecules.

Interestingly, when comparing the total detected areas obtained with the pD8 and PDMS fibers depicted in Figure 6a, it can be seen that they are very similar. However, when the individual compounds areas are considered in Figure 6b, distinct behaviors can be observed: for smaller alcohols and ketones, pD8 fiber showed the best results; for 1-octanol similar results were obtained for the two fibers; for monoterpenes, PDMS fiber showed higher detections than pD8. This might indicate that, even for compounds that have high water solubility such as benzyl alcohol, 3-pentanone, and 1-butanol, and thus lower headspace concentrations, pD8 showed higher sensitivity. Moreover, even pD2 fiber achieved higher sensitivities for these polar compounds than the two commercial fibers.

### 2.6. Validation, Matrix Effect and Recovery Using Fiber pD14

The analytical performance of the most efficient fiber, pD14, is represented in Table S2. As shown, large linear regions were obtained, with correlation coefficients varying from 0.987 to 0.999. Comparing the limits of detection (LODs) of pD14 with those of the PDMS7, it can be observed that they vary from analyte to analyte, in the range of 2 × 10^2^ nanograms for the lowest LODs, where pD14 showing always lower LODs than PDMS7. When comparing the overall LODs obtained for pD14 with those reported in the literature for other PIL-based fibers [12,38,39], they fall in the same detection limits range. As for fiber’s reproducibility, they were studied using two different situations: using the same fiber to extract similar solutions in different days, and extracting the same solution with different fibers. In terms of the total detectable area, 6% and 17% difference was obtained for the first and second case, respectively. The recovery studies were performed using pD14 fiber in human urine samples. Since no signals of the compounds under study were detected in the urine sample, recoveries were accessed by performing extractions from the urine sample spiked at two different analytes concentrations, 65 mg∙L^−1^ and 6.5 mg∙L^−1^. Recoveries ranging from 72.0% to 104.1% for the sample with the highest analyte concentration, and from 60.2% to 93.5% for the sample with the lowest analyte concentration, were obtained.

## 3. Materials and Methods

### 3.1. Synthesis of Ionic Liquid Monomers

Synthesis of diallylmethylalkylammonium bis (trifluoromethanesulfonyl)imide:

The synthesis of the diallylmethylalkylammonium bromide monomers ([DAMC_m_][Br] m = 2, 8 and 14) and diallylmethylalkylammonium bis (trifluoromethanesulfonyl)imide ([DAMC_m_][TFSI] m = 2, 8 and 14) are depicted in Figure 1a,b. [DAMC_m_][Br] (m = 2, 8 and 14) were prepared via a nitrogen-quaternization step (Figure 1a) where diallylmethylamine was mixed with an equivalent molar amount of the respective bromoalkane (C_m_Br, m = 2, 8 and 14) and left to react during 48 h at 60 °C in acetonitrile (ACN). The products were dried under nitrogen (N_2_) and washed three times with ethyl acetate (EA) (n = 1 and 7) and diethyl ether (DEE) (n = 13). After drying under N_2_, yellowish powders were obtained for n = 7 and 13 and whitish powder for n = 1. [DAMC_m_][Br] (m = 2, 8 and 14) purities were accessed by ^1^H-NMR on a Bruker 400 MHz Ultra-Shield-Plus Magnet NMR instrument.

For the anion exchange reaction (Figure 1b), a mixture containing an equimolar amount of each one of [DAMC_m_][Br] (m = 2, 8, and 14) and lithium bis(trifluoromethanesulfonyl)imide salt (LiTFSI) was left to react during 12 h in methanol (MeOH) at room temperature. Later, the products were dried under N_2_ and washed several times with water to ensure the complete removal of salts. The products were then dried under N_2_ and vacuum (around 2 × 10^−3^ mbar). Afterwards, they were dissolved in acetone (ACT), and if no precipitate was present, the products were dried under vacuum again and stored at 4 °C for future use. The resulting monomers are viscous brownish liquids for n = 7 and 13, and a transparent liquid for n = 1.

### 3.2. TGA and DSC Experiments

For the TGA and DSC experiments, the polymeric materials were prepared as films by using a mixture composed of the monomer of interest, divinylbenzene (DVB) as crosslinker (25% in weight relative to monomer), and 2-hydroxy-2-methylpropiophenone as photoinitiator (PI) (5% in weight relative to monomer). Each mixture was transferred into a silicon mold and placed under the UV light (Dymax ECE Flood Lamp 5000 equipped with a metal halide bulb—UVA, Dymax, Torrington, CT, USA) to induce polymerization. The thermal stabilities and decomposition temperatures of the prepared materials were measured using a TGA Q50, while the glass transition temperatures were determined using a DSC Q200 differential scanning calorimeter, both from TA Instruments (TA Instruments, New Castle, DE, USA).

The TGA experiments were performed in aluminum pans and under N_2_ atmosphere using the following temperature program: (1) heating from room temperature up to 250 °C at 10 °C·min^−1^; (2) cooling to room temperature; (3) heating from room temperature to 250 °C at 10 °C·min^−1^; (4) isothermal at 250 °C during 30 min; and (5) heating up to 500 °C at 10 °C·min^−1^.

Prior to DSC experiments, the prepared films were placed in an oven at a temperature of 200 °C during 30 min. Then, the films were transferred to aluminum pans and under N_2_ atmosphere, the following DSC temperature program was used: (1) cooling from room temperature to −70 °C; (2) heating from −70 °C to 210 °C, and then; (3) cooling from 210 °C to −70 °C, at a heating/cooling rate of 1 °C·min^−1^. Three cycles were performed for each sample.

### 3.3. SPME Fiber Coating Preparation

The fiber fabrication was based on a methodology proposed by Zeng et al. [24], which is illustrated in the Figure 3. The process starts by vertically immersing 1 cm of a fused silica tube (ST) with 0.34 mm of internal diameter (Figure 3a) in a beaker containing the prepared mixture (monomer, crosslinker and photoinitiator (M1)). By capillary forces, M1 enters the ST, completely filling it (Figure 3b). Then, a steel wire (SW) with approximately 0.2 mm external diameter was cleaned with MeOH, ACT, hexane (HEX), and dichloromethane (DCM) to remove any contamination, and then dried in an oven during 12 h for the complete removal of any solvent. This SW was carefully inserted in the center of the ST filled with the M1 solution. Two rubber stoppers were placed on both ends of the ST (Figure 3c) and this assembly was then placed under the UV light to induce photopolymerization (Figure 3d). After 60 s, the assembly was taken from the UV chamber and it was dipped in a 40% hydrofluridic acid (HF) solution during 1 h to dissolve the ST (Figure 3e). The resulting coated fiber was placed in MeOH to remove any reactants left and some HF and in the end a homogenous polymer coating on top of the SW was obtained, that it is referred as fiber from here onwards (Figure 3f).

Finally, the prepared fiber was glued to a 500 μL Hamilton syringe plunger and conditioned at 200 °C during 1 h. All of the fibers were analyzed by scanning electron microscopy (SEM) Hitachi S2400 (Hitachi High-Technologies Europe GmbH, Krefeld, Germany) with Bruker light elements EDS detector (Bruker Corporation, Billerica, MA, USA) (Figure 4).

### 3.4. Headspace—SPME Methodology

The Headspace (HS) SPME extraction of standard aqueous solutions composed of DL-menthol, (1R)-(+)-camphor (*S*)-(−)-β-citronellol; 3-pentanone, 2-hexanone, cyclopentanone, 2-heptanone; 1-butanol, 2-heptanol, 1-octanol, and benzyl alcohol, was carried out using the homemade coated fibers along with commercial fibers for comparison purpose. All fibers were preconditioned prior their first use and conditioned during 10 min, at recommended specific temperature, before their daily use. For the SPME procedure, 4 mL of each standard solution were transferred into 10 mL glass vials and the HS—SPME parameters such as stirring rate, extraction temperature, salt addition or extraction time were controlled depending on the desired study condition. After extraction, the fibers were immediately inserted in the GC injector and kept there for 3 min. Between each set of experiments, a blank injection was used to confirm the absence of analytes from previous extractions (carryover). All of the experiments were done in triplicate to estimate uncertainties. The analytical performance and efficiency of each SPME fiber coating was carried out in a Trace 1300 gas chromatograph (GC) from Thermo Scientific (Thermo Fisher Scientific, Waltham, MA, USA) equipped with a flame ionization detector (FID). Helium was used as carrier gas at 1.5 mL·min^−1^. The GC injector was maintained at 200 °C with a splitless time of 0.2 min and FID was maintained with a temperature of 250 °C. A TG-17MS (30 m × 250 μm × 0.25 μm) capillary column from Thermo Scientific was used with the following temperature program: initial temperature of 35 °C during 3 min, followed by a ramp at 20 °C·min^−1^ to 100 °C, held during 3 min, and then increased to 280 °C at a ramp of 25 °C min^−1^.

### 3.5. Matrix Effect, Validation and Recovery Tests

The analytical performances, such as linearity, of the Pyrr-PIL fibers were evaluated using the following conditions: *T* = 45 °C, *t* = 15 min, 2.5 wt % of NaCl and standard solution concentrations varying from 0 to 0.2 g·L^−1^. Limits of detection were determined by decreasing the concentration of each one of the standard solutions until the signal to noise ratio of 3 (S/N = 3) was achieved. The recovery tests were accessed by using a real human urine sample. The urine sample was collected from a healthy 31 years old man, filtered using a 0.45 μm pore size PTFE filter, and stored at 4 °C. Relative recoveries were obtained by spiking known amounts of analytes into the urine sample.

## 4. Conclusions

The development of pyrrolidinium-based polymeric ionic liquids for solid phase microextraction fibers is here presented for the first time. Three different polymers with different alkyl side chain lengths of two, eight, and fourteen carbons were synthesized and used as dense SPME fibers. A one step, solvent free, simple, and time saving polymerization and fiber preparation strategy, through the use of UV irradiation, was successfully demonstrated. The results obtained for a model mixture of analytes from different chemical families indicate that the PIL-based fibers are well suited for the model solution, being the polymer with longer alkyl chain length the better performing one, in terms of detection capability, due to its larger free volume. A comparison with the commercially available fibers based on Polyacrylate and Polydimethylsiloxane allows us to conclude that the PIL-based fibers displayed a superior performance. Good recoveries were obtained when studying a urine sample, ranging from 60.2% to 104.1%. Therefore, further research on poly(ionic liquid) materials for development of analytical tools that allow for the detection of very low concentration of diverse compounds is of great interest. The inherent tunability of this class of materials will be very valuable in the detection of specific families of compounds.

## Supplementary Materials

The following are available online at www.mdpi.com/1996-1944/10/9/1094/s1, Figure S1: ^1^H-NMR data example of the synthesized diallylmethyloctyl bromide ([DAMC_8_] Br) ionic liquid monomer.; Figure S2: Thermo gravimetric analysis data of the crosslinked poly(methyloctyl pyrrolidinium) TFSI (pD8) under N_2_ atmosphere.; Figure S3: Thermo gravimetric analysis data of the crosslinked poly(methylethyl pyrrolidinium) TFSI (pD2) under N_2_ atmosphere.; Figure S4: Differential Scanning Calorimetry data of the crosslinked poly(methyltetradecyl pyrrolidinium) TFSI (pD14).; Figure S5: Differential Scanning Calorimetry data of the crosslinked polymers. From top to bottom: pD14, pD8 and pD2.; Table S1: Glass transition temperatures and decomposition temperatures obtained for the three crosslinked polymers. Table S2: Representation of the linear range and limits of detection for the analytes under study for the pD14 fiber. (*T* = 45 °C, *t* = 15 min, 2.5 wt % of NaCl, 200 rpm).
