# Supplementary material for: Expanding the Applicability of Poly(Ionic Liquids) in Solid Phase Microextraction: Pyrrolidinium Coatings
Source: Materials (Basel). 2017 Sep 18;10(9):1094. doi: 10.3390/ma10091094 (PMC5615748; doi:10.3390/ma10091094)
Supplement: Supplementary file 1 [file materials-10-01094-s001.pdf]

Supporting information

Expanding the applicability of poly(ionic liquids) in solid phase microextraction: pyrrolidinium coatings

David J. S. Patinha,[1,2] Liliana C. Tomé,[1] Mehmet Isik,[3] David Mecerreyes,[3,4] Armando J. D. Silvestre,[2*] and Isabel M. Marrucho,[1,2*]

1 Instituto de Tecnologia Química e Biológica António Xavier, Universidade Nova de Lisboa, Av. Da República, 2780-157, Oeiras, Portugal; davidpatinha@itqb.unl.pt (DJSP), liliana.tome@itqb.unl.pt (LCT)
2 CICECO - Aveiro Institute of Materials and Department of Chemistry, University of Aveiro, 3810-193 Aveiro, Portugal; armsil@ua.pt (AJDS)
3 POLYMAT, University of the Basque Country UPV/EHU, Joxe Mari Korta Center, Avda. Tolosa 72, 20018 Donostia-San Sebastian, Spain; isik.mehmet@ehu.eus (MI), david.mecerreyes@ehu.es (DM)
4 IKERBASQUE, Basque Foundation for Science, E-48011 Bilbao, Spain
5 Centro de Química Estrutural, Instituto Superior Técnico, Universidade de Lisboa, Avenida Rovisco Pais, 1049-001 Lisboa, Portugal; isabel.marrucho@tecnico.ulisboa.pt (IMM)
* Correspondence: armsil@ua.pt, Tel: +351 234 370711; isabel.marrucho@tecnico.ulisboa.pt; Tel: +351-21-4469724; fax: +351-21-4411277

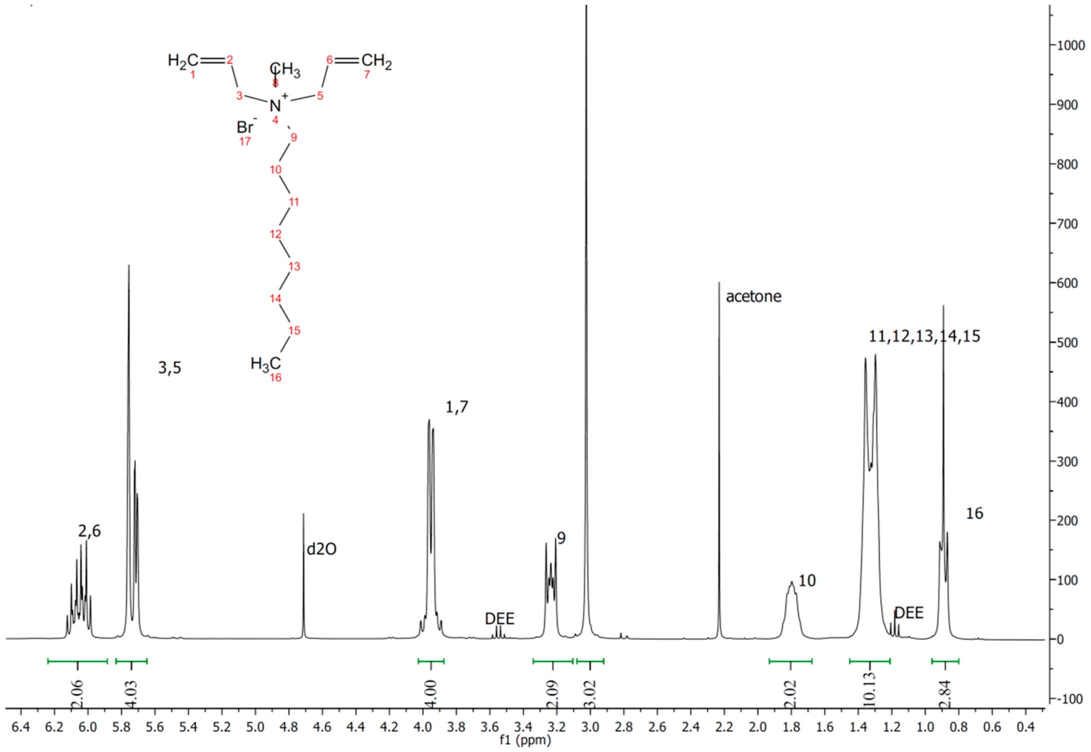

Figure S1 –^{1}H NMR data example of the synthesized diallylmethyloctyl bromide ([DAMC$_8$] Br) ionic liquid monomer. H^1 NMR assignments data for the three monomers: **[DAMC$_{14}$][Br]** - ^{1}H NMR (400 MHz, deuterated dimethyl sulfoxide (*d_6*-DMSO)): δ 0.67-0.89 (t, 3H), δ 1.01-1.37 (m, 22H), δ 1.56-1.81 (q, 2H), δ 3.06-3.22 (s, 3H), δ 3.22-3.37 (t, 2H), δ 4.05-4.29 (d, 4H), δ 5.56-5.70 (dd, 4H) and δ 5.84-6.05 (m, 2H). **[DAMC$_8$][Br]** - ^{1}H NMR (400 MHz, Deuterium oxide (*D_2O*)): δ 0.80-0.95 (t, 3H), δ 1.20-1.40 (m, 10H), δ 1.70-1.91 (q, 2H), δ 2.90-3.10 (s, 3H), δ 3.15-3.25 (t, 2H), δ 3.90-4.10 (d, 4H), δ 5.70-5.85 (d, 4H) and δ 5.90-6.20 (m, 2H). **[DAMC$_2$][Br]** - ^{1}H NMR (400 MHz, *D_2O*): δ 1.25-1.35 (t, 3H), δ 2.85-2.95 (s, 3H), δ 3.25-3.35 (q, 3H), δ 3.75-3.90 (d, 4H), δ 5.56-5.70 (d, 4H) and δ 5.84-6.05 (m, 2H).

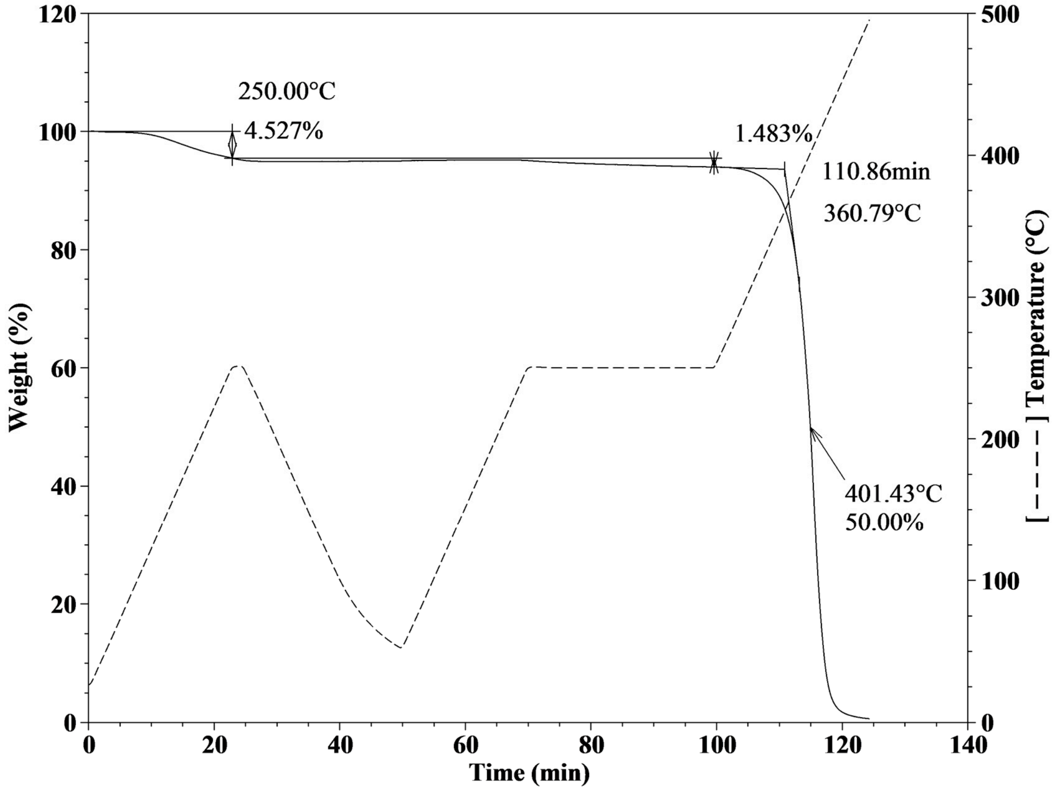

Figure S2 – Thermo gravimetric analysis data of the crosslinked poly(methyloctyl pyrrolidinium) TFSI (pD8) under N_2 atmosphere.

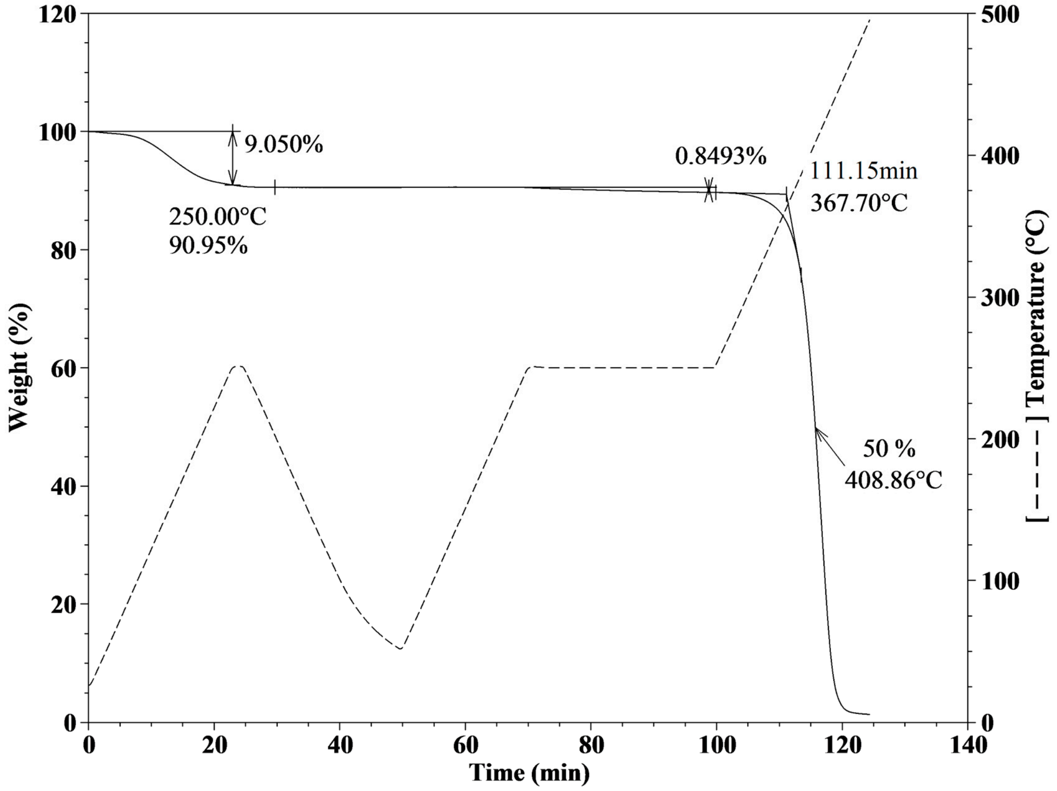

Figure S3 – Thermo gravimetric analysis data of the crosslinked poly(methylethyl pyrrolidinium) TFSI (pD2) under N_2 atmosphere.

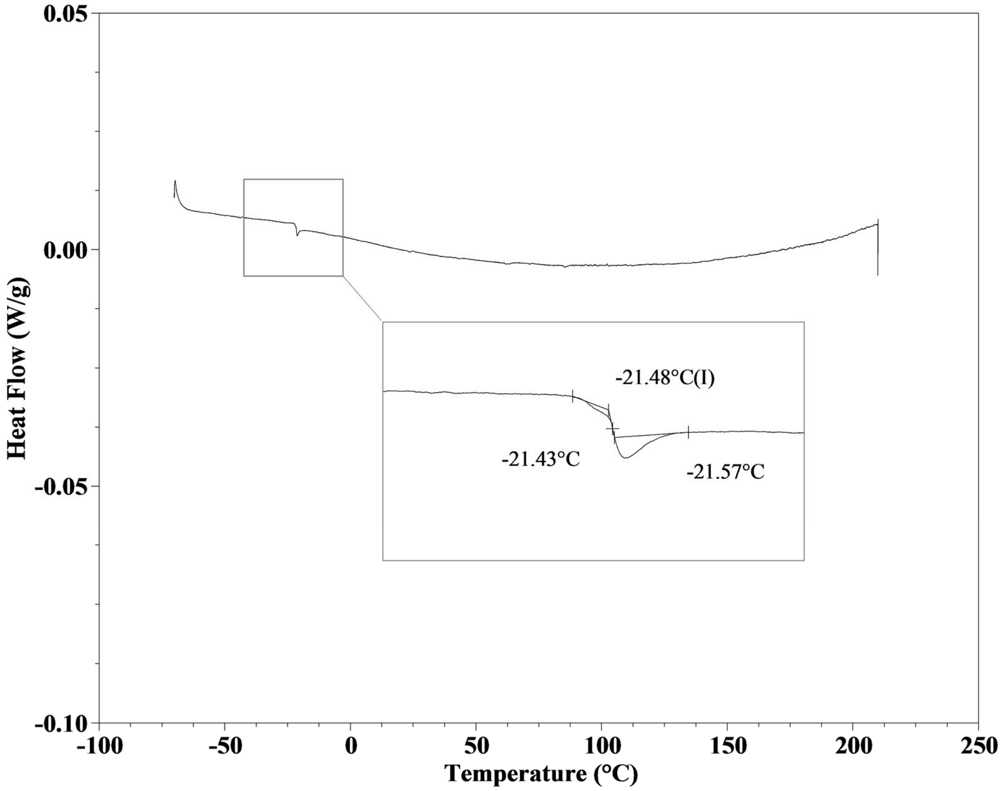

Figure S4 – Differential Scanning Calorimetry data of the crosslinked poly(methyltetradecyl pyrrolidinium) TFSI (pD14).

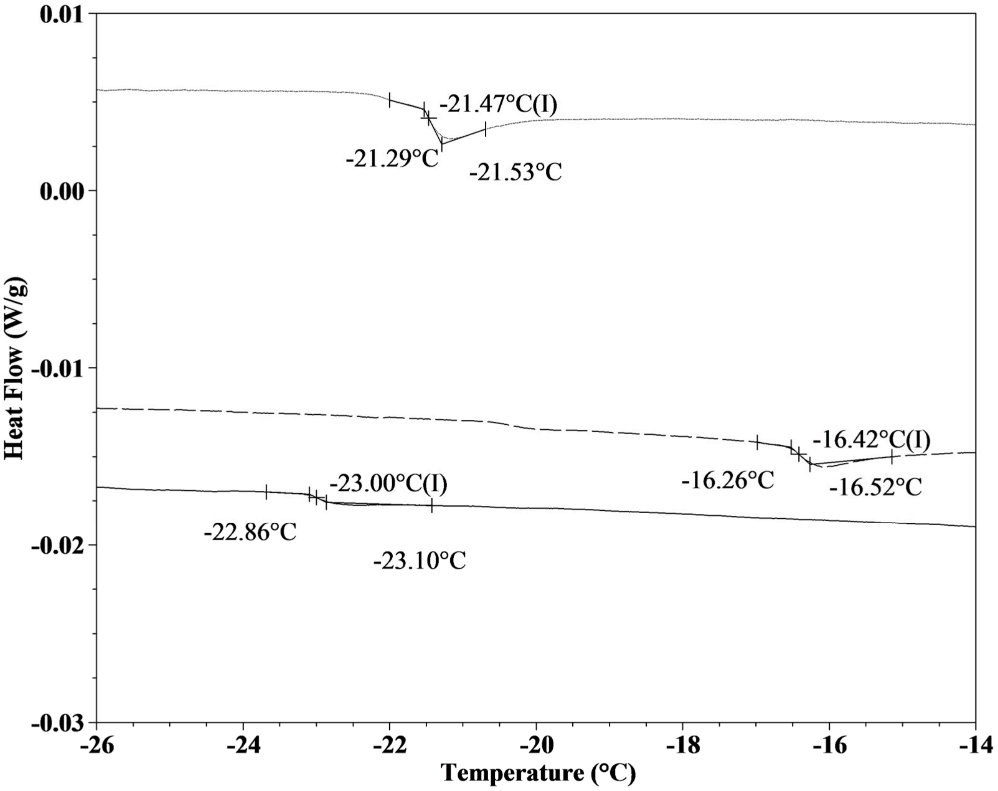

Figure S5 – Differential Scanning Calorimetry data of the crosslinked polymers. From top to bottom: pD14, pD8 and pD2.

Table S1 – Glass transition temperatures and decomposition temperatures obtained for the three crosslinked polymers.

Polymers	T_g / ºC	T_d / ºC
pD14	-21.47	368
pD8	-16.42	361
pD2	-23.00	355

Table S2 – Representation of the linear range and limits of detection for the analytes under study for the pD14 fiber. (T = 45 ºC, t = 15 min, 2.5 wt. % of NaCl, 200 rpm).

Sample name	***Linear range μg·L⁻¹ (×10³)***	***r***	***slope***	***LOD μg·L⁻¹***	
				pD14	PDMS
1-butanol	5-100	0.999	55±0.7	200	200
3-pentanone	2.5-100	0.989	281±11	200	200

2-hexanone	0.02-100	0.993	595±15	2	2
cyclopentanone	2.5-100	0.991	72±2.4	200	200
2-heptanol	0.25-100	0.996	387±7.9	5	20
2-heptanone	0.02-100	0.994	1332±29	1	2
1-octanol	0.02-100	0.993	1866±44	1	2
benzyl alcohol	0.5-100	0.999	134±1.2	20	50
DL-menthol	0.25-100	0.987	1044±32	0.2	2
(1R)-(+)-camphor	0.02-100	0.998	441±5.8	0.5	2
(S)-(-)-β-citronellol	0.02-100	0.999	1322±12	0.2	2
